# Liver steatosis and dyslipidemia after HCV eradication by direct acting antiviral agents are synergistic risks of atherosclerosis

**DOI:** 10.1371/journal.pone.0209615

**Published:** 2018-12-21

**Authors:** Naoki Kawagishi, Goki Suda, Akinobu Nakamura, Megumi Kimura, Osamu Maehara, Kazuharu Suzuki, Akihisa Nakamura, Masatsugu Ohara, Takaaki Izumi, Machiko Umemura, Masato Nakai, Takuya Sho, Mitsuteru Natsuizaka, Kenichi Morikawa, Koji Ogawa, Yusuke Kudo, Mutsumi Nishida, Hideaki Miyoshi, Naoya Sakamoto

**Affiliations:** 1 Department of Gastroenterology and Hepatology, Hokkaido University Graduate School of Medicine, Sapporo, Hokkaido, Japan; 2 Department of Rheumatology, Endocrinology and Nephrology, Faculty of Medicine and Graduate School of Medicine, Hokkaido University Graduate School of Medicine, Sapporo, Hokkaido, Japan; 3 Division of Laboratory and Transfusion Medicine, Hokkaido University Hospital, Sapporo, Hokkaido, Japan; 4 Division of Diabetes and Obesity, Faculty of Medicine and Graduate School of Medicine Hokkaido University, Sapporo, Hokkaido, Japan; Policlinico Universitario Campus Bio-Medico, ITALY

## Abstract

**Aim:**

We comprehensively analyzed how hepatitis C virus (HCV) eradication by interferon (IFN)-free direct-acting-antiviral-agents (DAAs) affects liver steatosis and atherogenic risk.

**Methods:**

Patients treated with IFN-free-DAAs who underwent transient elastography before and at 24-weeks post-treatment, including controlled attenuation parameter (CAP), and achieved sustained viral response (SVR) were enrolled. The association between changes in liver steatosis, lipid-metabolism, and genetic and clinical factors was analyzed.

**Results:**

A total of 117 patients were included. The mean CAP and low-density lipoprotein cholesterol (LDL-C) levels were significantly elevated at SVR24. However, baseline LDL-C and CAP values were significantly negatively correlated with changes in these values after HCV eradication, indicating that in patients with high baseline values, the values generally decreased after HCV eradication. Mean small-dense LDL-C (sdLDL-C), which has greater atherogenic potential, was significantly elevated only in patients with both dyslipidemia (LDL-C >140 mg/dL) and liver steatosis (CAP >248 dB/m) at SVR24. Those patients had significant higher baseline BMI, LDL-C, and total-cholesterol levels.

**Conclusions:**

Generally, successful HCV eradication by IFN-free-DAAs decreases CAP and LDL-C in patients with high baseline values. However, elevated LDL-C was accompanied with elevated sdLDL-C only in patients with liver steatosis and dyslipidemia at SVR24; therefore, those patients may require closer monitoring.

## Introduction

Hepatitis C virus (HCV) is one of the major pathogens causing liver cirrhosis and hepatocellular carcinoma (HCC) globally. Recently developed direct acting antiviral agents (DAAs) have dramatically changed the landscape of anti-HCV treatment. Various clinical trials and accumulated real-world data clearly reveal that interferon (IFN)-free DAAs regimens are safe and ensure high rates of sustained viral response (SVR) [1–6]. Large numbers of HCV-infected patients have been successfully cured by IFN-free DAA therapy, including patients at high risk of hepatocellular carcinogenesis, such as those with advanced liver fibrosis, liver steatosis, diabetes, and the elderly [7, 8]. In addition, HCV-infected patients with concomitant disease, including renal dysfunction, cardiovascular disease, and metabolic syndrome, have achieved successful HCV eradication [5]. Thus, it is necessary to clarify the effect of HCV eradication by IFN-free DAA therapy on hepatocellular carcinogenesis and concomitant diseases.

Hepatic steatosis is one of the characteristic histological finding in the livers of HCV-infected patients [9]. Overproduction of lipid droplets is observed in HCV-transfected cells [10]. HCV is known to utilize host lipid droplets as a scaffold for viral assembly [10]. These experimental results indicate that HCV recruits lipid droplets for its replication, resulting in hepatic steatosis. In terms of the host, hepatic steatosis causes insulin resistance, hepatic fibrosis, and oxidative stress [11, 12] and can lead to HCC [13, 14]. Several possible mechanisms of HCV-induced liver steatosis have been reported. HCV infection causes activation of sterol-regulatory-element-binding-protein (SREBP) 1c [15], which is a transcription factor involved in lipogenesis. HCV-related liver steatosis is also partially caused by a decrease in mitochondrial β-oxidation [16]. Carnitine palmitoyltransferase-1 (CPT-1), which a regulator of mitochondrial β-oxidation, is downregulated by HCV infection [17, 18].

Additionally, HCV core protein inhibits the activity of microsomal triacylglycerol transfer protein (MTP), which is essential and a rate-limiting factor in the assembly and secretion of very low-density lipoprotein-cholesterol (VLDL-C), resulting in liver steatosis and hypolipidemia [19].Thus, while successful HCV eradication is thought to ameliorate liver steatosis, it may cause elevation of VLDL-C and low-density lipoprotein-cholesterol (LDL-C). LDL-C plays a key role in the development and progression of atherosclerosis, leading to cardiovascular disease and cerebral infarction. LDL-C consists of large buoyant, intermediate, and small dense LDL-C (sdLDL-C). SdLDL-C has greater atherogenic potential and is a better marker for prediction of cardiovascular disease than LDL-C [20–22].

However, the actual changes in serum lipid profile and liver steatosis, and their relationship with successful HCV eradication by IFN-free DAAs have been not clarified well. This had been previously difficult to evaluate during the IFN therapy era due to IFN’s effect on lipoprotein disorders [23, 24].

The gold standard for liver steatosis diagnosis is liver biopsy. However, biopsy may cause various complications and is prone to sampling error. Recently, several non-invasive methods for the evaluation of liver fibrosis and steatosis have been developed. FibroScan (Echosens, Paris, France) can evaluate liver stiffness measurement (LSM) for liver fibrosis assessment, and controlled attenuation parameter (CAP) for liver steatosis with great accuracy [25].

Genetic factors, including patatin-like phospholipase domain-containing protein 3 (PNPLA3) and transmembrane 6 superfamily member 2 (TM6SF2) affect liver steatosis with or without dyslipidemia [26–28]. As previously mentioned, MTP has an important role in metabolizing hepatic triglycerides and VLDL-C for secretion from the liver and its variants were associated with an increased risk of liver steatosis [29]. However, the effect of genetic factors on liver steatosis and dyslipidemia after successful HCV eradication by IFN-free DAAs has been not well understood.

We aimed to comprehensively investigate the factors associated with liver steatosis and dyslipidemia after HCV eradication by IFN-free DAAs, which may cause HCC and atherosclerosis.

## Methods

### Patients and study design

In this retrospective study at Hokkaido University Hospital between October 2014 and November 2017, a total of 234 patients with HCV infection who received IFN-free DAAs therapy were screened. Inclusion criteria were: available clinical information, preserved serum samples, and paired FibroScan LSM for liver fibrosis assessment and CAP for liver steatosis assessment at baseline and SVR24 point. Patients were excluded if they had a history of liver transplantation, did not achieve SVR, were regularly taking lipid-lowering agents, had missing clinical information, or did not undergo paired FibroScan examination at baseline and the SVR24 point.

Among the screened patients, 117 who met the inclusion criteria were included in this study. We analyzed the changes in total cholesterol (T-C), LDL-C, high-density lipoprotein-cholesterol (HDL-C), LSM and CAP values, glycoalbumin (GA), body mass index (BMI), and laboratory data in all patients. In addition, 100 patients who agreed to genomic analysis (UMIN000031092) were screened for PNPLA3 (*rs738409*), MTP493 (*rs1800591*), and TM6SF2 (*rs58542926*) polymorphisms, which are associated with liver steatosis and/or dyslipidemia.

The study protocol conformed to the ethical guidelines of the Declaration of Helsinki and was approved by the ethics committee of Hokkaido University Hospital. Informed consent was obtained from all patients. This study was registered at the UMIN Clinical Trials Registry as UMIN000031091.

### Anti-HCV protocols

For daclatasvir (DCV) and asunaprevir (ASV) combination therapy, both DCV (60 mg, once daily) and ASV (100 mg, twice daily) were orally administered to patients with genotype 1 HCV infection for 24 weeks. For sofosbuvir (SOF) and ribavirin (RBV) combination therapy, both SOF (400 mg, once daily) and RBV were orally administered to patients with genotype 2 HCV infection for 12 weeks. RBV was administered according to body weight (patients ≤60 kg received 600 mg daily, 60–80 kg received 800 mg daily, and >80 kg received 1000 mg daily). For SOF and ledipasvir (LDV) combination therapy, a fixed-dose combination tablet containing SOF (400 mg) and LDV (90 mg) was orally administered once daily, to patients with genotype 1 HCV infection for 12 weeks. For ombitasvir/paritaprevir/ritonavir (OBV/PTV/r) combination therapy, a fixed-dose combination tablet containing OBV/PTV/r (25 mg/150 mg/100 mg) was orally administered once daily, to patients with genotype 1 HCV infection for 12 weeks.

### LSM and CAP

FibroScan 502 (Echosens) was used for measuring LSM and CAP with the M-probe and XL-probe. Each patient was placed in the supine position with the right hand at the most abducted position during the procedure. At least 10 valid measurements were obtained, and effective measurements were defined as those more than 60% and interquartile range less than 30%. Median values were adopted as the result.

### Single nucleotide polymorphism genotyping

Genomic DNA was extracted from the patients’ whole blood samples. A TaqMan single nucleotide polymorphism genotyping kit (Applied Biosystems, Foster City, CA) was utilized for analyzing PNPLA3 *rs738409* (C/G), MTP493 *rs1800591* (G/T), and TM6SF2 *rs58542926* (C/T). Genotyping was carried out according to the manufacturer's protocol a using a Step One Plus Real-Time PCR System (Applied Biosystems). The overall genotype completion rate was 99.7%.

### Examination of small dense LDL-C

SdLDL-C was measured using enzymatic kits obtained from Denka Seiken (Tokyo, Japan).

### Statistical analyses

Continuous variables were analyzed with the paired Mann-Whitney *U*-test, the Wilcoxon test, or one-way analysis of variance as appropriate. Categorical data were compared using the Chi-squared test. We selected the optimal cut-off point on the receiver operating characteristics (ROC) curve by maximizing the Youden index. The relationship between two variables was assessed using Spearman’s rank correlation. All *P*-values were two-tailed, and the level of significance was set at *P* <0.05. All statistical analyses were performed using SPSS version 24.0 (IBM Japan, Tokyo, Japan).

## Results

### Patients

We screened 234 patients with HCV infection who received IFN-free DAA therapy between October 2014 and November 2017. Of those 234 patients, 117 who had undergone paired FibroScan examination at baseline and the SVR24 points and had available clinical information and preserved serum were included in this study (**S1 Fig**). The baseline characteristics of enrolled patients are shown in **S1 Table**. Of 117 enrolled patients, 21, 51, 38, and 7 were treated with DCV plus ASV, SOF plus LDV, SOF plus RBV, and OBV plus PTV/r, respectively. The patients were 22–85 years of age (median, 64 years), and 53.8% (63/117) were female. The baseline median BMI, T-C, HDL-C, and LDL-C were 22.4 kg/m^2^ (range, 15.6–30.9), 171 mg/dL (68–278), 51 mg/dL (21–131), and 93 mg/dL (19–197), respectively. The median LSM and CAP value were 6.8 kPa (3.1–37.5) and 214 dB/m (100–343), respectively. In addition, of 117 patients, 100 patients had available information on PNPLA3 *rs738409* and TM6SF2 *rs58542926* genotyping. During MTP493 *rs1800591* genotyping, a valid result was not obtained in 1 of the 100 patients.

### Changes in lipid profile, CAP, GA, LSM, and body weight after successful HCV eradication

**Fig 1:**
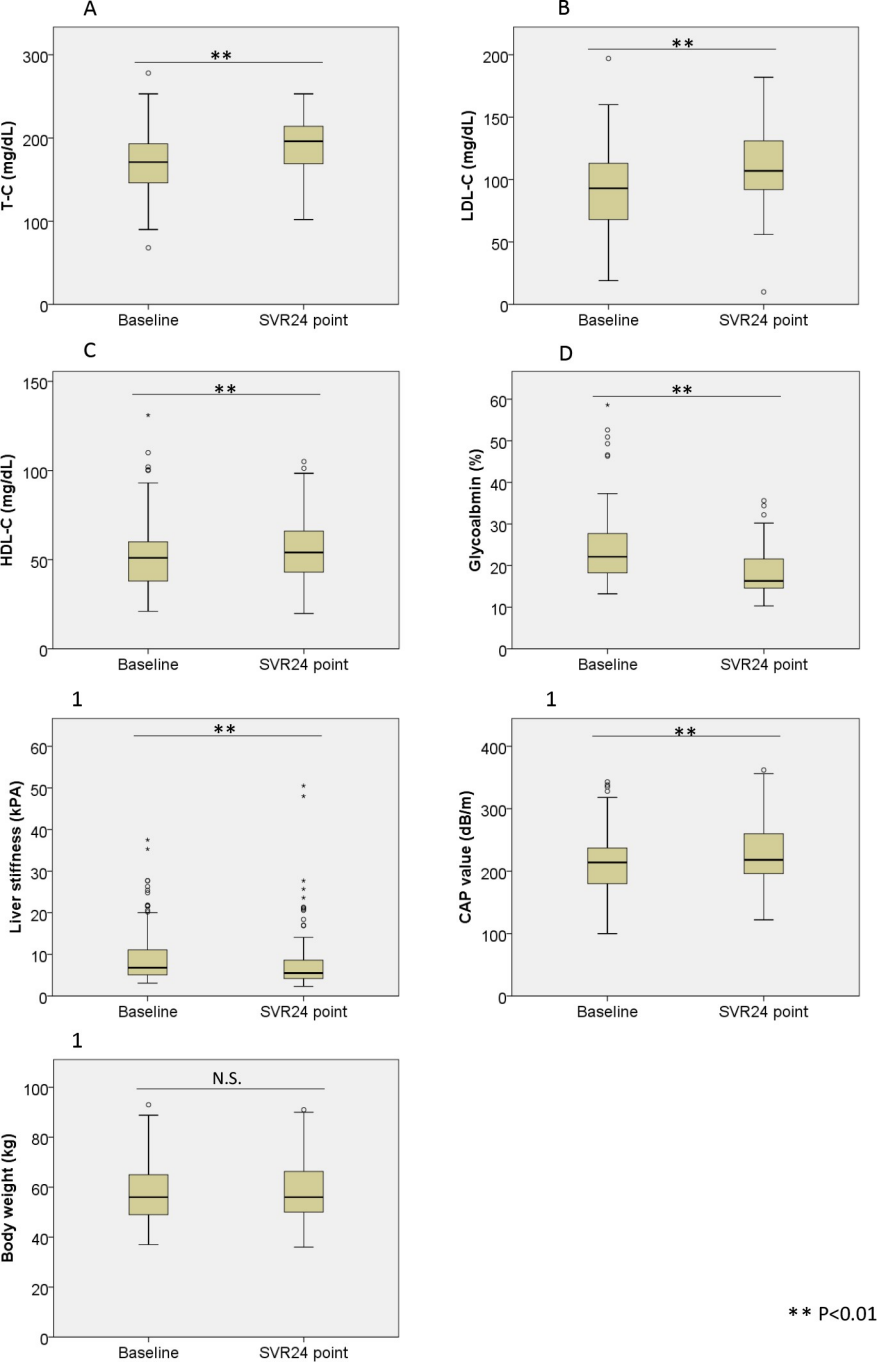
Changes in lipid profile, body weight, glycoalbumin, LS, and CAP after successful HCV eradication by IFN-free DAAs. Changes in (1A) total-cholesterol (T-C), (1B) low density lipoprotein-cholesterol (LDL-C), (1C) high density lipoprotein-cholesterol (HDL-C), (1D) glycoalbumin, (1E) liver stiffness (LS), (1F) controlled attenuation parameter (CAP), and (1G) body weight after successful HCV eradication by IFN-Free DAAs. T-C, LDL-C, HDL-C, and CAP were significantly elevated at the SVR24 point compared with baseline. Conversely, glycoalbumin and liver stiffness were improved, and body weight did not significantly change. Statistically significant difference, ** P <0.01. N.S, not significant; LS, liver stiffness; CAP, controlled attenuation parameter; DAAs, direct-acting antiviral agents.

We evaluated the changes in T-C, LDL-C, HDL-C, GA, LSM, CAP values, and body weight between baseline and the SVR24 point. As shown in **Fig 1A–1G**, the mean T-C, LDL- C, HDL-C, and CAP were significantly elevated at the SVR24 point. By contrast, GA and LSM were significantly decreased and body weight did not significantly change.

### Correlation between baseline CAP and change in CAP after successful HCV eradication

**Fig 2:**
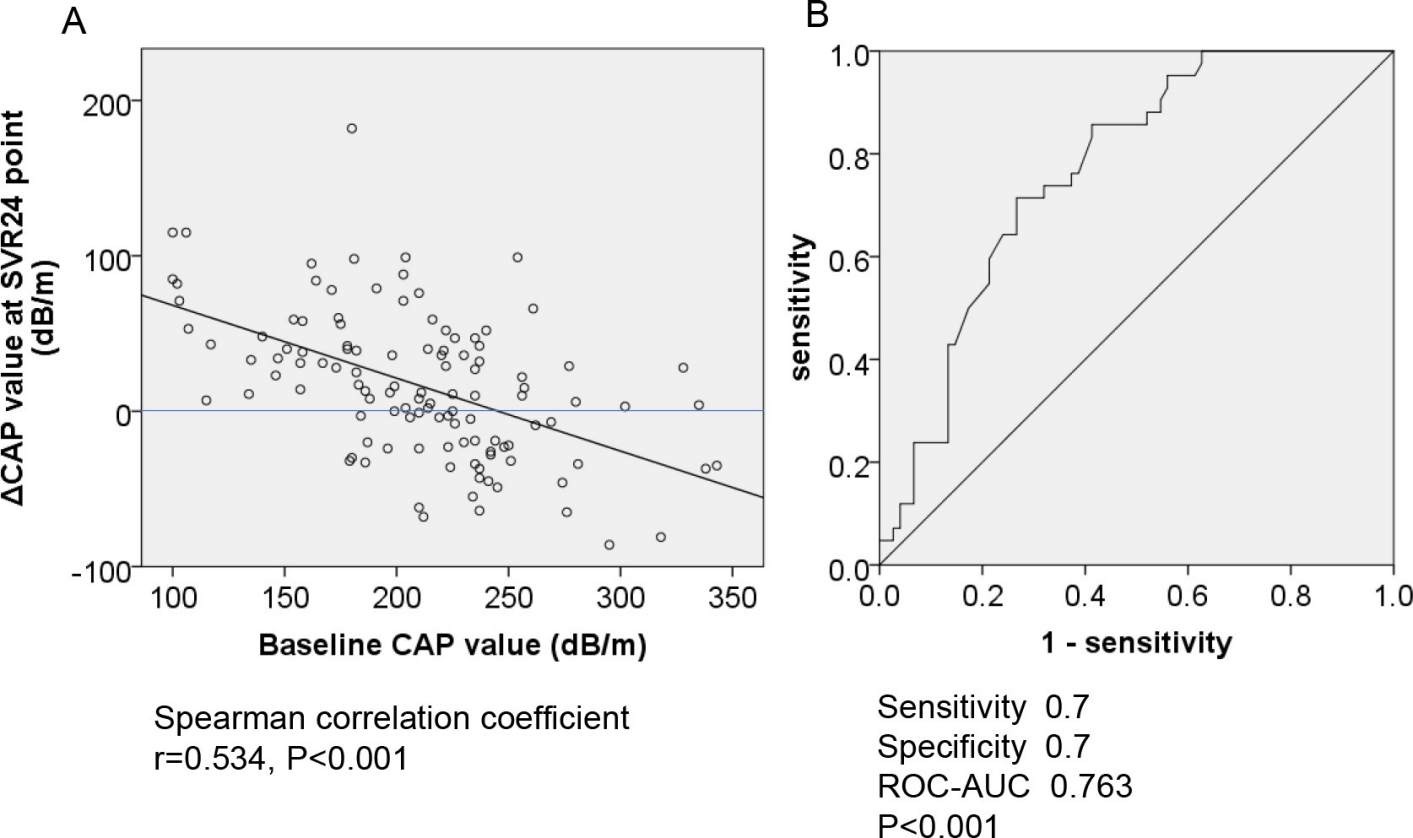
A-B. The correlation between baseline CAP and the change in CAP and after successful HCV eradication by IFN-free DAAs. (2A) The baseline CAP value and ΔCAP values were significantly negatively correlated (r = 0.534, P <0.001). (2B) Receiver operating characteristics (ROC) curve analysis for ΔCAP value. The cut off baseline CAP associated with ΔCAP was 220 dB/m (ROC-AUC = 0.763; P <0.001; sensitivity, 0.7; specificity, 0.7). CAP; controlled attenuation parameter, ΔCAP; the value at SVR24 minus the value at baseline, SVR; sustained viral response, DAAs, direct-acting antiviral agents.

Next, we analyzed the associations between baseline CAP and its change after HCV eradication. As shown in **Fig 2A**, the baseline CAP value and the change in CAP values were significantly negatively correlated. Subsequently, we conducted ROC analysis to determine the cut off baseline CAP value associated with decrease of CAP value after HCV eradication. As shown in **Fig 2B**, the cut off value was set at 220 dB/m (sensitivity, 0.7; specificity, 0.7; ROC-AUC, 0.763; *P* <0.001). This indicated that, although CAP significantly increased after HCV eradication across the entire cohort, patients with baseline CAP >220 dB/m experienced decreased CAP after HCV eradication. The baseline characteristics of patients with or without baseline CAP >220 dB/m is shown in **S2 Table**. Next, we examined the characteristics of patients with baseline CAP >220 dB/m that experienced CAP value decreases after HCV eradication. As shown in **S3 Table**, the patients who experienced decreased CAP had a significantly lower baseline BMI.

### Correlation between baseline LDL-C and change in LDL-C after successful HCV eradication

**Fig 3:**
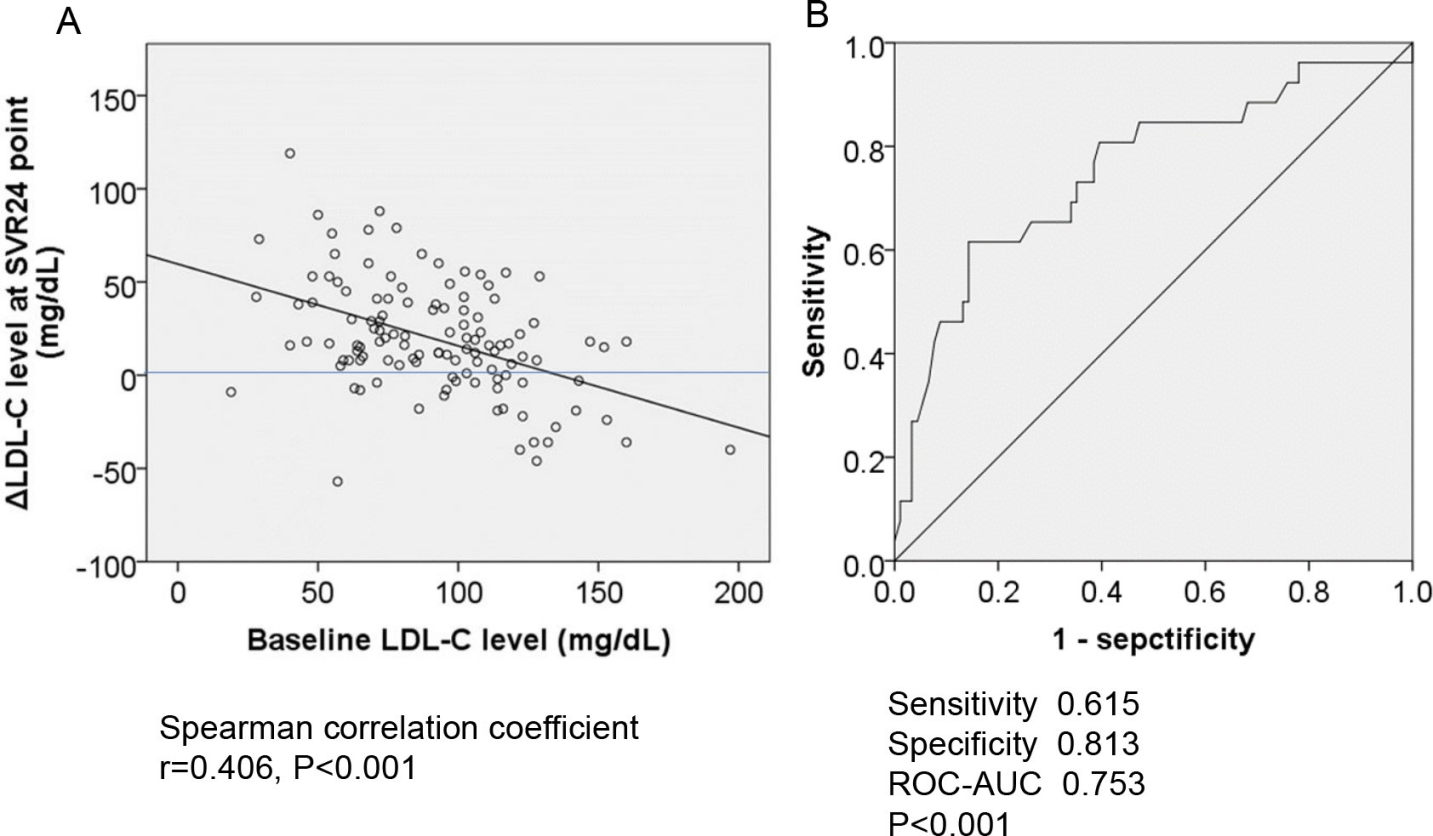
A-B. The correlation between baseline LDL-C and the change in LDL-C after successful HCV eradication by IFN-free DAAs. (3A) The baseline LDL-C level and ΔLDL-C level were significantly negatively correlated (r = 0.406, P <0.001). (3B) ROC curve analysis for ΔLDL-C level. The cut off baseline LDL-C associated with ΔLCL-C is 108 mg/dL (ROC-AUC = 0.615; P <0.001; sensitivity, 0.813; specificity, 0.753). LDL-C; low density lipoprotein-cholesterol, ΔLDL-C; the value at SVR24 minus the value at baseline, SVR; sustained viral response, DAAs, direct-acting antiviral agents.

Subsequently, we analyzed the associations between baseline LDL-C and the changes in LDL-C after HCV eradication. As shown in **Fig 3A,** baseline LDL-C levels and their changes were significantly negatively correlated. Subsequently, we conducted ROC analysis to determine the cut off values of baseline LDL-C level associated with decreases after HCV eradication. As shown in **Fig 3B**, the cut off value was set at 108 mg/dL (sensitivity, 0.615; specificity, 0.813; ROC-AUC, 0.753; *P* <0.001). This indicated that although LDL-C levels were significantly increased after HCV eradication in whole-cohort analysis, patients with baseline LDL-C >108 mg/dL experienced decreases. A comparison of the baseline characteristics of patients with or without baseline LDL-C >108 mg/dL is shown in **S4 Table**. Next, we examined the characteristics of patients with baseline LDL-C >108 mg/dL who experienced LDL-C decreases. As shown in **S5 Table**, the patients who experienced decreased LDL-C levels after HCV eradication had significantly higher baseline LDL-C and lower BMI compared with patients who had increased LDL-C.

### The association between liver steatosis and serum lipid level after successful HCV eradication

**Fig 4:**
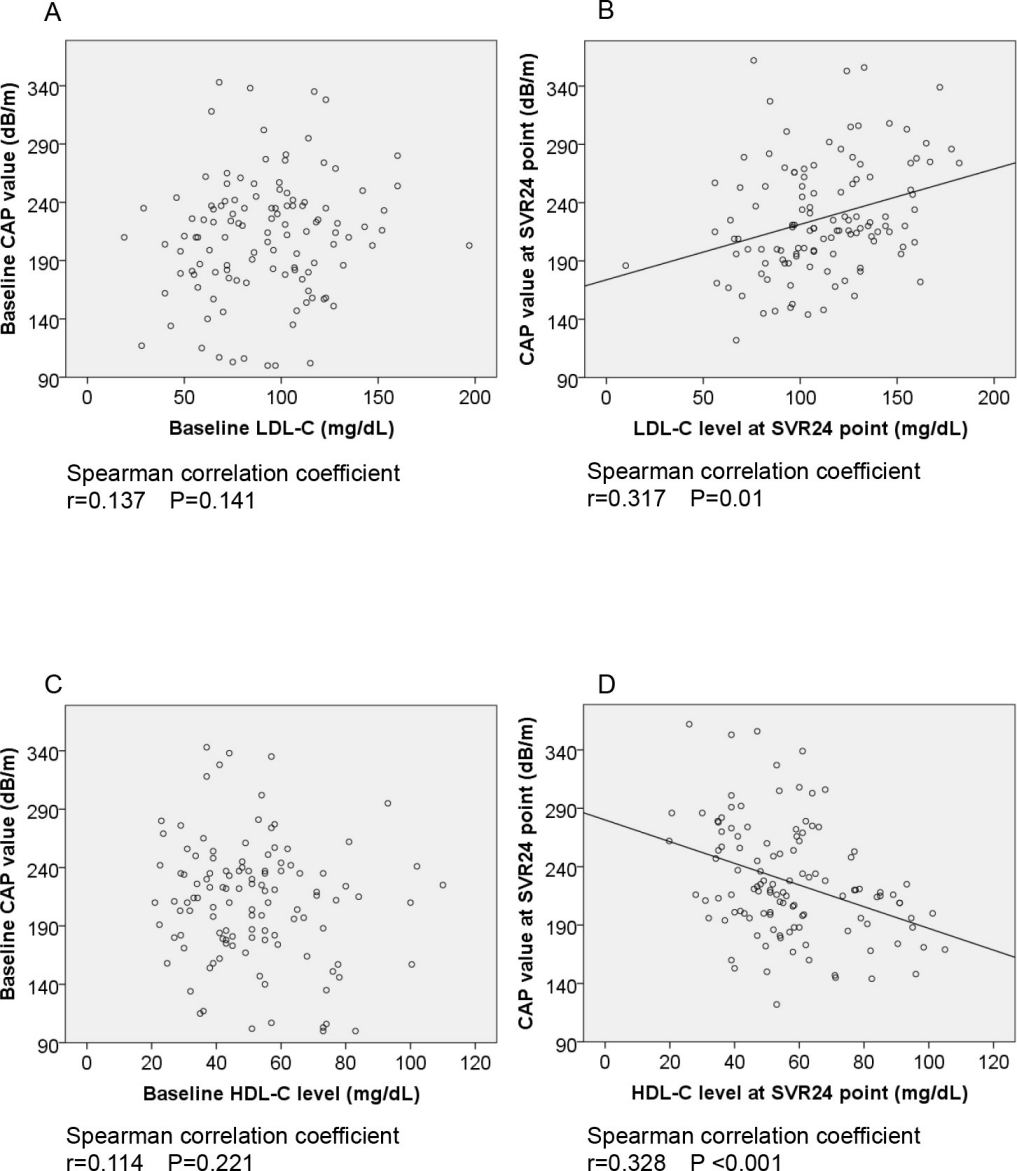
A-D. The association between liver steatosis and serum cholesterol levels after successful HCV eradication by IFN-free DAAs. (4A) At baseline, there was no significant correlation between CAP and LDL-C (r = 0.137; P = 0.141). However, (4B) at the SVR24 point there was a significant correlation (r = 0.317, P = 0.01). (4C) At baseline (existence of HCV status), there was not significant correlation between CAP value and HDL-C level (r = 0.114, P = 0.221); however, (4D) at SVR24 point, significant correlation between CAP value and HDL-C level (r = 0.328, P <0.001). CAP, controlled attenuation parameter; LDL-C, low density lipoprotein-cholesterol; HDL-C, high density lipoprotein-cholesterol; SVR, sustained viral response; DAAs, direct-acting antiviral agents.

Subsequently, we evaluated the association between CAP value and LDL-C or HDL-C levels at baseline and the SVR24 point. As shown in **Fig 4A and 4B**, there was no significant correlation between CAP value and LDL-C level at baseline; however, after successful HCV eradication, a significant correlation was observed. Similarly, a significantly negative correlation between CAP value and HDL-C level was only observed after HCV eradication (**Fig 4C and 4D)**.

### Risk factors of significant liver steatosis and/or dyslipidemia after successful HCV eradication

**Fig 5:**
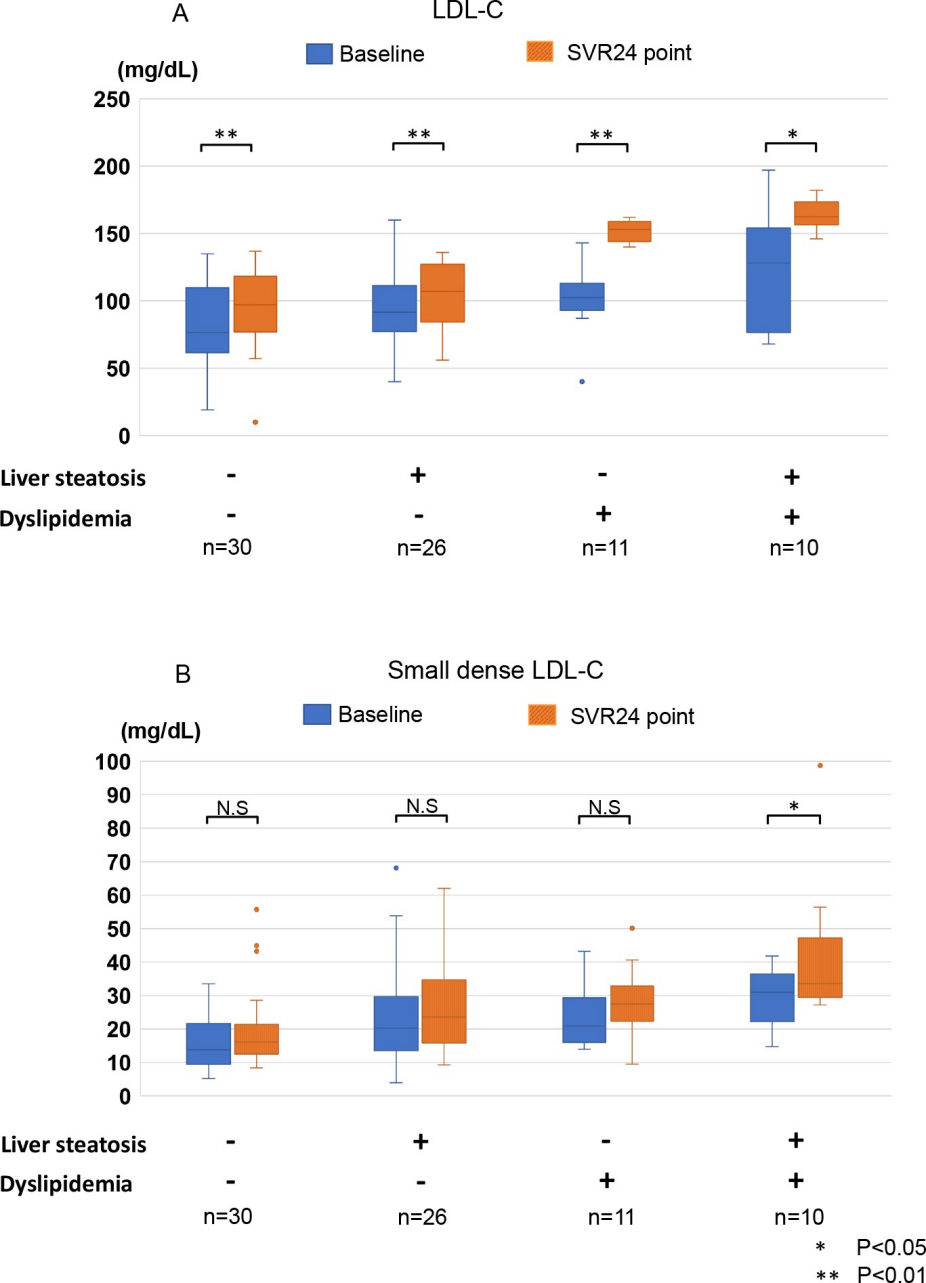
A-B. Changes in LDL-C and sdLDL-C after successful HCV eradication by IFN-free DAAs. The changes in LDL-C (5A) and sdLDL-C (5B) after HCV eradication by IFN-free DAAs were compared among 4 subgroups according to CAP and LDL-C at the SVR24 point (CAP < 248dB/m and LDL-C <140 mg/dL as controls; CAP >248 dB/m and LDL-C <140 mg/dL; CAP < 248dB/m and LDL-C > 140mg/dL; and CAP >248 dB/m and LDL-C >140 mg/dL). The boxplots demonstrate each lipid’s levels at baseline and the SVR24 point. Significant differences from the control group and between baseline and the SVR24 point were observed (*, P <0.05; **, P <0.01). Liver steatosis, CAP >248 dB/m; dyslipidemia, LDL-C > 140 mg/dL; LDL-C, low density lipoprotein-cholesterol; sdLDL-C, small dense LDL-C; CAP, controlled attenuation parameter; SVR, sustained viral response; DAAs, direct-acting antiviral agents.

We subsequently analyzed the factors associated with dyslipidemia and/or significant liver steatosis at the SVR24 point. Dyslipidemia (LDL-C >140 mg/dL) was defined according to the diagnostic criteria for screening of the Japan Atherosclerosis Society Guidelines for Prevention of Atherosclerotic Cardiovascular Diseases [30]. CAP >248 dB/m, indicating >10% prevalence of hepatocytes with fat (S1), was used to identify significant liver steatosis[25]. As shown in the **Table 1,** higher baseline BMI and CAP values and lower HDL-C levels were significantly associated with CAP >248 dB/m at the SVR24 point. Meanwhile, higher baseline T-C and LDL-C levels and lower liver stiffness were significantly associated with LDL-C >140 mg/dL at the SVR24 point (**Table 2**). Finally, we compared the changes in sdLDL-C, which have been reported to be strongly correlated with development and progression of atherosclerosis and cardiovascular disease [22, 31], in all patients with CAP >248 dB/m and/or LDL-C >140 mg/dL, subgroups according to CAP and LDL-C, and 30 control patients. As shown in **Fig 5A**, all subgroups experienced significant elevation of mean LDL-C level after HCV eradication. The subgroup of patients with CAP >248 dB/m and LDL-C >140 mg/dL had significantly higher sdLDL-C at the baseline and SVR24 points than the control group (**Fig 5B**) and experienced a significant increase between baseline and SVR24. Thus, this subgroup of patients may be at an increased risk of HCC and cardiovascular disease after HCV eradication. As shown in **Table 3,** patients with both CAP >248 dB/m and LDL-C >140 mg/dL at the SVR24 point had significant higher baseline BMI, LDL-C, and T-C levels.

**Table 1:** Comparison of baseline characteristics between patients with or without CAP >248 dB/m at the SVR24 point.

	CAP <248 dB/m	CAP ≧248 dB/m	P value
Number	79	38	
Age (years) ^†^	66 (22–85)	61.5 (35–83)	0.075
Sex (male/female)	33/46	21/17	0.17
HCV-RNA (log IU/mL) ^†^	6.2 (3.6–7.1)	6.3 (3.2–7.2)	0.676
BMI (kg/m^2^) ^†^	20.6 (15.63–29.96)	24.61 (19.37–30.86)	*<0.001
Baseline ALT (IU/L) ^†^	36 (6–273)	54.5 (11–262)	0.07
Baseline Fib-4 index^†^	3.01 (0.54–82.8)	2.65 (0.59–9.12)	0.526
Baseline T-C (mg/dL) ^†^	169 (68–278)	174 (92–247)	0.528
Baseline HDL-C (mg/dL) ^†^	53 (21–131)	42 (22.4–81)	*0.005
Baseline LDL-C (mg/dL) ^†^	93 (19–153)	95 (29–197)	0.135
Baseline Liver stiffness (kPa) ^†^	6.8 (3.1–26.3)	6.45 (3.7–37.5)	0.963
Baseline CAP (dB/m) ^†^	199 (100–318)	235 (162–343)	*<0.001
Baseline GA (%) ^†^	22.1 (13.2–52.6)	21.9 (14.1–58.6)	0.918
Genotype: number (n = 100)	68	32	
MTP493 GG/GT/TT	47/18/2	18/11/3	0.246
TM6SF2 CC/CT/TT	56/12/0	28/4/0	0.513
PNPLA3 CC/CG/GG	20/34/14	15/11/6	0.211

Abbreviations: HCV, Hepatitis C virus; BMI, body mass index; ALT, alanine aminotransferase; T-C, total-cholesterol; HDL-C, high density lipoprotein-cholesterol; LDL-C, low density lipoprotein-cholesterol; CAP, controlled attenuation parameter; GA, glycoalbumin. MTP493, microsomal triacylglycerol transfer protein 493; TM6SF2, transmembrane six superfamily member 2; PNPLA3, patatin-like phospholipase domain-containing protein 3.

^†^Data are shown as median (range) values.

*Statistically significant difference, P <0.05.

**Table 2:** Comparison of baseline characteristics between patients with or without LDL-C >140 mg/dL at the SVR24 point.

	LDL-C <140 mg/dL	LDL-C ≧140 mg/dL	P value
Number	96	21	
Age (years) ^†^	65 (22–85)	62 (35–76)	0.203
Sex (male/female)	45/51	9/12	0.465
HCV-RNA (log IU/mL) ^†^	6.3 (3.2–7.2)	6 (4.6–6.8)	0.283
BMI (kg/m^2^) ^†^	22.07 (16.58–30.86)	23.42 (15.63–29.21)	0.147
Baseline ALT (IU/L) ^†^	38 (6–262)	54 (14–273)	0.296
Baseline Fib-4 index^†^	3.02 (0.54–82.8)	2.42 (0.59–13.51)	0.081
Baseline T-C (mg/dL) ^†^	169 (68–278)	193 (134–247)	*0.002
Baseline HDL-C (mg/dL)^†^	51 (21–131)	51 (23–84)	0.812
Baseline LDL-C (mg/dL) ^†^	85.5 (19–169)	110 (40–197)	*0.002
Baseline Liver stiffness (kPa)^†^	6.9 (3.1–37.5)	5.9 (3.3–15.3)	*0.039
Baseline CAP (dB/m)^†^	211.5 (100–338)	216 (100–343)	0.223
Baseline GA (%) ^†^	22 (13.2–58.6)	22.9 (17.2–46.6)	0.659
Genotype: number (n = 100)	83	17	
MTP493 GG/GT/TT	53/26/4	12/3/1	0.597
TM6SF2 CC/CT/TT	70/13/0	14/3/0	0.541
PNPLA3 CC/CG/GG	29/37/17	6/8/3	0.962

Abbreviations: HCV, Hepatitis C virus; BMI, body mass index; ALT, alanine aminotransferase; T-C, total-cholesterol; HDL-C, high density lipoprotein-cholesterol; LDL-C, low density lipoprotein-cholesterol; CAP, controlled attenuation parameter; GA, glycoalbumin. MTP493, microsomal triacylglycerol transfer protein 493; TM6SF2, transmembrane six superfamily member 2; PNPLA3, patatin-like phospholipase domain-containing protein 3.

^†^Data are shown as median (range) values.

*Statistically significant difference, P <0.05.

**Table 3:** Comparison of baseline characteristics between patients with or without both CAP value >248 dB/m and LDL-C level >140 mg/dL at the SVR24 point.

	CAP >248 dB/m and LDL-C level >140 mg/dL	others	P value
Number	10	107	
Age (years)^†^	62 (35–76)	64 (22–85)	0.31
Sex (male/female)	4/6	50/57	0.473
HCV-RNA (log IU/mL) ^†^	5.85 (4.7–6.8)	6.3 (3.2–7.2)	0.467
BMI (kg/m^2^) ^†^	24.47 (20.32–29.21)	22.08 (15.63–30.86)	*0.023
Baseline ALT (IU/L) ^†^	45.5 (14–93)	40 (6–273)	0.785
Baseline Fib-4 index ^†^	1.92 (0.59–6.33)	2.91 (0.54–82.8)	0.073
Baseline T-C (mg/dL) ^†^	197.5 (153.6–247)	169 (68–278)	*0.007
Baseline HDL-C (mg/dL) ^†^	40 (23–71)	51 (21–131)	0.137
Baseline LDL-C (mg/dL) ^†^	128 (68–197)	92 (19–160)	*0.006
Baseline Liver stiffness (kPa) ^†^	5.1 (3.8–15.3)	6.8 (3.1–37.5)	0.08
Baseline CAP (dB/m) ^†^	222 (203–343)	211 (100–338)	0.064
Baseline GA (%)^†^	23.5 (17.7–46.6)	21.9 (13.2–58.6)	0.365
Genotype: number (n = 100)	8	93	
MTP493 GG/GT/TT	5/3/0	60/26//5	0.701
TM6SF2 CC/CT/TT	7/1/0	77/15/0	0.624
PNPLA3 CC/CG/GG	4/2/2	31/43/18	0.487

Abbreviations: HCV, Hepatitis C virus; BMI, body mass index; ALT, alanine aminotransferase; T-C, total-cholesterol; HDL-C, high density lipoprotein-cholesterol; LDL-C, low density lipoprotein-cholesterol; CAP, Controlled Attenuation Parameter; GA, glycoalbumin. MTP493, microsomal triacylglycerol transfer protein 493; TM6SF2, transmembrane six superfamily member 2; PNPLA3, patatin-like phospholipase domain-containing protein 3.

^†^Data are shown as median (range) values.

*Statistically significant difference, P <0.05.

## Discussion

Previous reports clearly showed that lipids play a key role in the HCV life cycle, and lipid metabolism is manipulated by HCV during replication [10]. HCV infection causes upregulation of SREBP 1c, which is associated with lipogenesis [15], and downregulation of MTP [9] and CPT-1, which are essential for the assembly and secretion of VLDL-C and regulation of mitochondrial β-oxidation [17, 18], resulting in liver steatosis. Therefore, eradication of HCV by IFN-free DAA therapy is expected to down-regulate SREBP 1c and up-regulate MTP and CPT-1, resulting in a decrease in lipogenesis in the liver and an increase in VLDL secretion. Thus, CAP value, which is a surrogate marker of liver steatosis, is thought to decrease. However, in our study, overall CAP values were significantly elevated at the SVR24 point compared with those at baseline (**Fig 1F**), which is consistent with recent reports [32, 33]. Further analysis revealed that the change in CAP at the SVR24 point was negatively correlated with the baseline value. This indicated that while most patients with less severe liver steatosis at baseline experienced elevation of CAP, patients with higher baseline experienced a decrease. Thus, the baseline liver steatosis was ameliorated after HCV eradication. However, some patients with baseline CAP >220 dB/m experienced an elevation at the SVR24 point, and those patients had a significantly higher BMI. In addition, as shown in **S6 Table**, when baseline CAP was <220 dB/m, some patients experienced a remarkable elevation after HCV eradication, exceeding 248dB/m at the SVR24 point, and had a significantly higher baseline BMI and lower HDL-C level than the others. Since significant liver steatosis is an independent risk factor for HCC after HCV eradication [7, 34], and Tanaka et al had reported that most patients with post-eradication HCC had liver steatosis at diagnosis [35], careful monitoring is needed in patients with post-eradication steatosis.

Recent reports showed that successful HCV eradication by IFN-free DAA therapy causes elevation of LDL-C [36, 37]. HCV infection cause hypocholesterolemia [38]; therefore, successful treatment may lead to elevation of cholesterol, including LDL-C. Similarly, in this study, the mean LDL-C and T-C levels were significantly elevated after successful eradication. By contrast, as shown in S2 Fig, in patients with non-SVR by IFN-free DAAs, LDL-C level did not change after DAA treatment. However, we are the first to show that baseline LDL-C levels and their changes at the SVR24 point were significantly negatively-correlated. More specifically, in patients with LDL-C >108 mg/dL, LDL-C levels generally tended to decrease. The precise mechanisms underlying this observation are unclear. Serum LDL-C levels are modulated by the synthesis and exertion of VLDL-C, and the uptake of LDL-C via the LDL receptor (LDL-R) in hepatocytes. Previous reports showed that in HCV infected patients, LDL-R was significantly decreased [39, 40]. Therefore, change in the expression of LDL-R may be involved in the decrease in LDL-C after HCV eradication. However, further analysis is required.

Serum LDL-C levels are associated with atherosclerosis and cardiovascular events. The Japan Atherosclerosis Society Guidelines for Prevention of Atherosclerotic Cardiovascular Diseases define dyslipidemia as LDL-C >140 mg/dL. Therefore, we analyzed the factors associated with LDL-C >140 mg/dl at the SVR24 point. As shown in **Table 2**, lower baseline liver stiffness and higher LDL-C and T-C levels were significantly associated with this LDL-C threshold. Hypolipidemia is a feature of liver cirrhosis [41] and patients with advanced liver fibrosis, may have lower LDL-C levels at the SVR24 point due to deficient lipogenesis. As shown in **Fig 1B**, LDL-C levels were generally elevated after HCV eradication. Several previous reports had revealed that HCV infection is a risk factor for atherosclerosis due to the HCV-induced insulin resistance and inflammatory cytokine release [42]. HCV eradication by IFN therapy may thus reduce the risk of development and progression of atherosclerosis and cardiovascular events [43]. However, whether the same effect would be observed after DAA therapy in patients with elevated LDL-C remained unclear. Therefore, we analyzed the changes in sdLDL-C, which is more atherogenic potential and a better predictor cardiovascular disease than other LDL-C subfractions [44]. As shown in the **Fig 5B**, subgroup of patients with CAP >248 dB/m and LDL-C >140 mg/dL at the SVR24 point had significantly higher sdLDL-C than the control group, both at baseline and the SVR24 point. Importantly, only this subgroup experienced significant elevation of sdLDL-C. In contrast, the remaining subgroups all experienced significant elevation of LDL-C but not sdLDL-C.

The patients with LDL-C >140 mg/dL and CAP >248 dB/m at the SVR24 point had significantly higher baseline BMI, T-C, and LDL-C (**Table 3**). Thus, in patients with high BMI and higher LDL-C level at baseline, more careful monitoring for possible development of atherosclerosis or HCC is needed, regardless of HCV eradication.

Finally, we investigated how genetic factors affect these changes. We analyzed PNPLA3 *rs738409* (C/G), MTP493 *rs1800591* (G/T), and TM6SF2 *rs58542926* (C/T), which are known to be associated with liver steatosis/nonalcoholic steatohepatitis and/or lipid metabolism. As shown in **S7 and S8 Tables**, the PNPLA3 *rs738409* (C/G) and TM6SF2 *rs58542926* (C/T) genotypes demonstrated no effects on liver steatosis and dyslipidemia at baseline and SVR24. MTP493 *rs1800591* (G/T) was significantly associated with baseline T-C (**S9 Table)**. Thus, these genetic factors did not have a profound effect on changes in the lipid profile or liver steatosis. However, longer observation periods might be required to determine any effects after HCV eradication.

There are several limitations to our study. First this was a retrospective single-center study and with a relatively small sample size. In addition, the included DAA protocols were not unified. Therefore, a larger prospective study is required. In addition, in this study, liver steatosis was evaluated according to the CAP value, not histologically. Although several reports have shown the accuracy of CAP value in evaluating liver steatosis, further analysis, including histology, is required.

In conclusion, HCV eradication by IFN-free DAA therapy resulted in significant elevation of CAP and LDL-C values. However, successful HCV eradication by IFN-free DAAs decreased CAP and LDL-C in patients with higher baseline values and elevated LDL-C without an accompanied elevation of sdLDL-C, except in patients with liver steatosis and dyslipidemia at the SVR24 point. Therefore, those patients may require closer monitoring for HCC or atherosclerosis development and progression, regardless of HCV eradication. Prospective data with long-term follow-up is needed to prove such a hypothesis.

## Supporting information

S1 Fig Study design. (TIF)

S2 Fig Changes in LDL-C and sdLDL-C after non-SVR by IFN-free DAAs. (A) Changes in LDL-C levels between baseline and post 24 weeks after IFN-free DAA completion in patients with non-SVR (n = 11). (B) Changes in sdLDL-C level between baseline and post 24 weeks after IFN-free DAA completion in patients with non-SVR (n = 9). LDL-C, low density lipoprotein-cholesterol; sdLDL-C, small dense LDL; SVR, sustained viral response; DAAs, direct-acting antiviral agents; Post 24w, post 24 weeks after DAAs treatment completion.
(TIF)

S1 Table Baseline characteristics of patients. (DOCX)

S2 Table Comparison of baseline characteristics between patients with or without baseline CAP >220 dB/m. (DOCX)

S3 Table Comparison of baseline characteristics between patients with CAP > 220 dB/m that did or did not experience a decrease in CAP after HCV eradication. (DOCX)

S4 Table Comparison of baseline characteristics between patients with or without baseline LDL-C >108 mg/dL. (DOCX)

S5 Table Comparison of baseline characteristics between patients with LDL-C >108 dB/m that did or did not experience a decrease in LDL-C after HCV eradication. (DOCX)

S6 Table Comparison of baseline characteristics between patients with CAP <220 dB/m that did or did not exceed CAP >248 dB/m at SVR24 point. (DOCX)

S7 Table Association among changes in clinical parameters after HCV eradication according to the PNPLA3 genetic polymorphisms. (DOCX)

S8 Table Association among changes in clinical parameters after HCV eradication according to the TM6SF2 genetic polymorphisms. (DOCX)

S9 Table Association among changes in clinical parameters after HCV eradication according to the MTP-493 genetic polymorphisms. (DOCX)
