# Supplementary figures and images for: Liver steatosis and dyslipidemia after HCV eradication by direct acting antiviral agents are synergistic risks of atherosclerosis
Source: PLoS One. 2018 Dec 21;13(12):e0209615. doi: 10.1371/journal.pone.0209615 (PMC6303061; doi:10.1371/journal.pone.0209615)

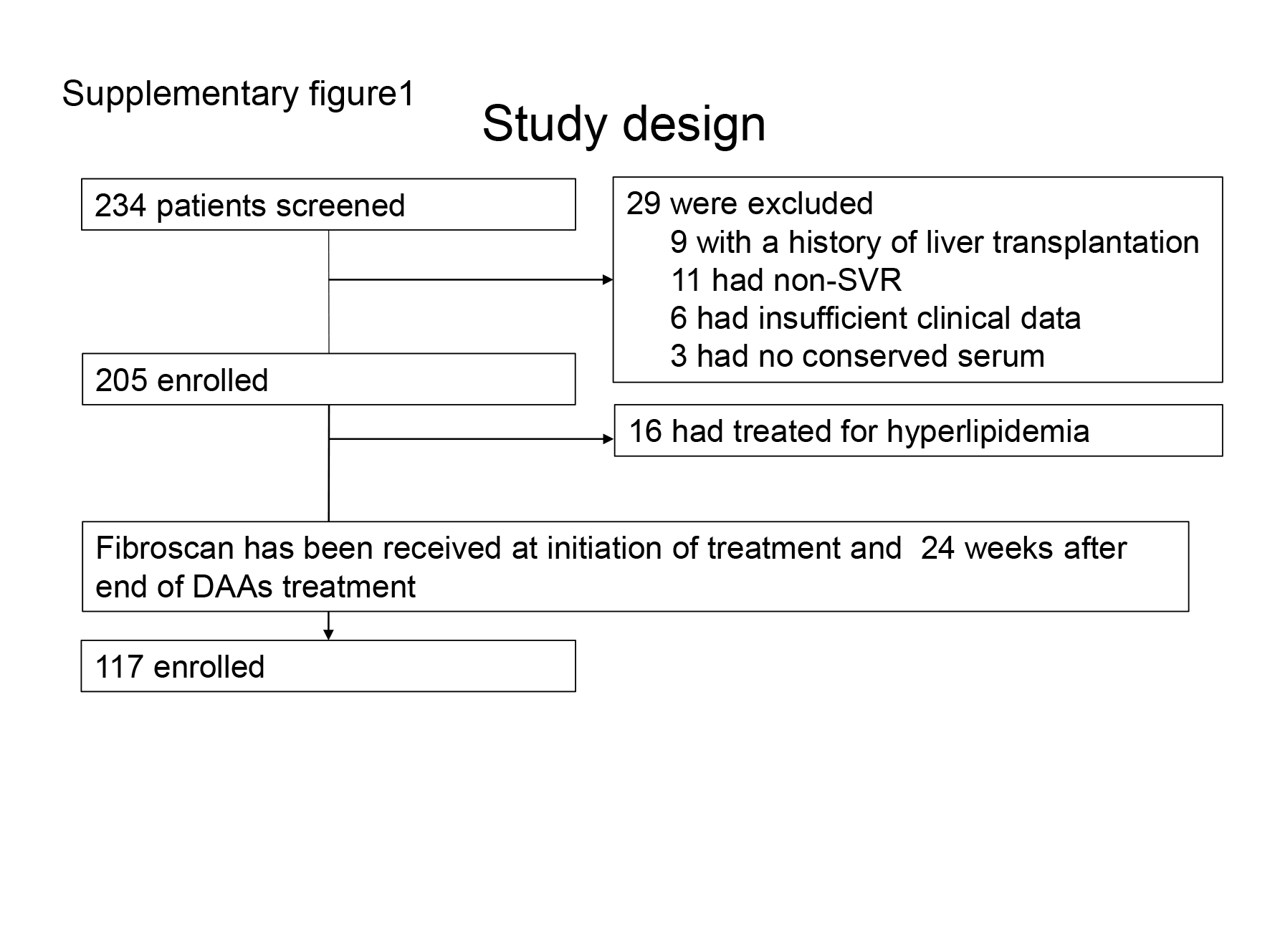

Supplement: S1 Fig — (TIF) [file pone.0209615.s001.tif]

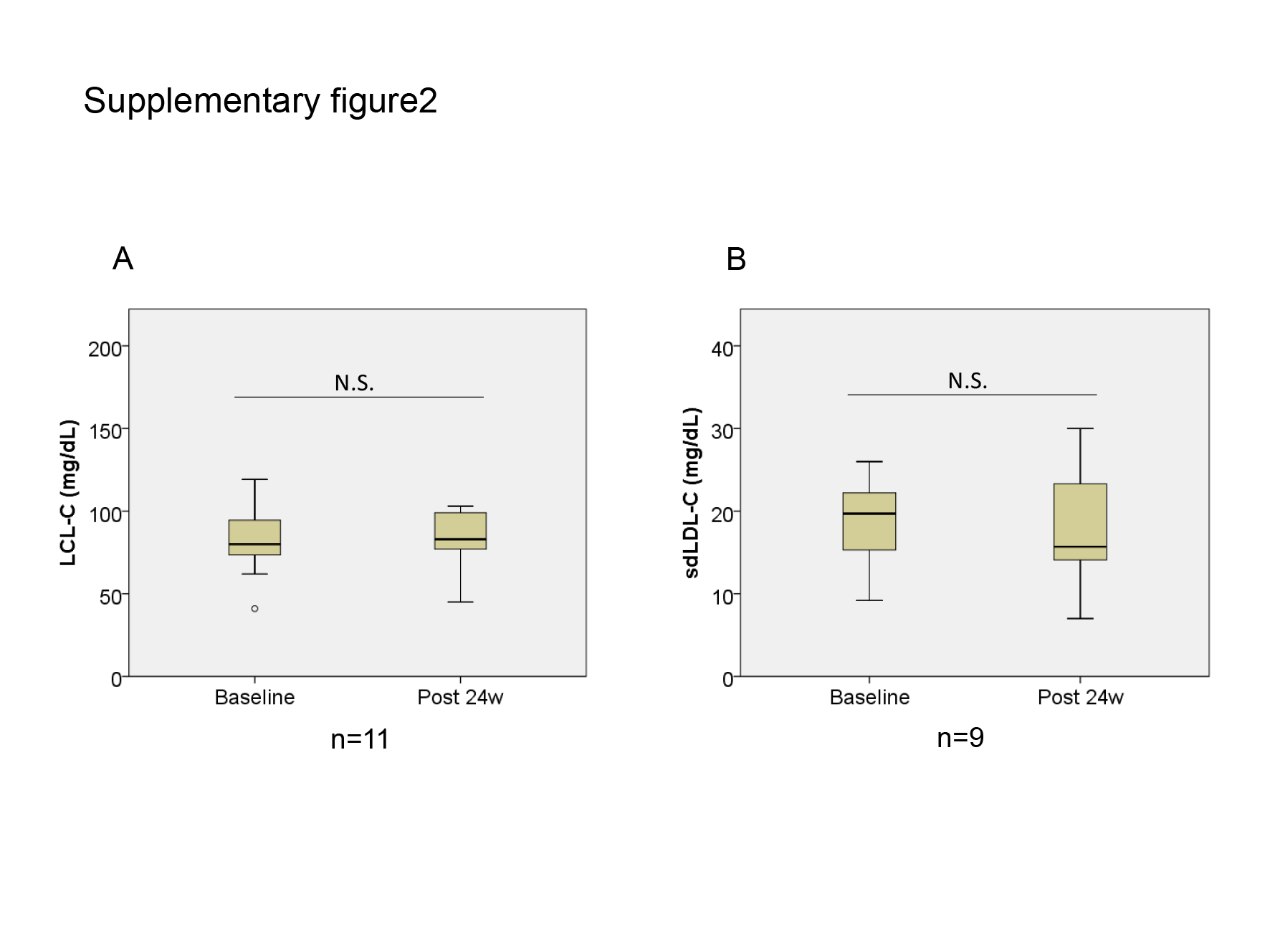

Supplement: S2 Fig — (A) Changes in LDL-C levels between baseline and post 24 weeks after IFN-free DAA completion in patients with non-SVR (n = 11). (B) Changes in sdLDL-C level between baseline and post 24 weeks after IFN-free DAA completion in patients with non-SVR (n = 9). LDL-C, low density lipoprotein-cholesterol; sdLDL-C, small dense LDL; SVR, sustained viral response; DAAs, direct-acting antiviral agents; Post 24w, post 24 weeks after DAAs treatment completion. (TIF) [file pone.0209615.s002.tif]
