# Supplementary material for: Liver steatosis and dyslipidemia after HCV eradication by direct acting antiviral agents are synergistic risks of atherosclerosis
Source: PLoS One. 2018 Dec 21;13(12):e0209615. doi: 10.1371/journal.pone.0209615 (PMC6303061; doi:10.1371/journal.pone.0209615)
Supplement: S1 Table — (DOCX) [file pone.0209615.s003.docx]

**Supplementary table 1 Baseline characteristics of patients**

|  | Median (range) |
| --- | --- |
| Number | 117 |
| Age (years) | 64 (22-85) |
| Sex (male/female) | 54/63 |
| Genotype 1/2 | 79/38 |
| DCV, ASV /SOF, LDV / SOF, RBV/ OBV, PTV, r | 21/51/38/7 |
| HCV-RNA (log IU/mL) | 6.3 (3.2-7.2) |
| BMI (kg/m^2^) | 22.4 (15.6-30.9) |
| Baseline Platelet count (×10^4^) | 15.2 (0.7-37.3) |
| Baseline Albumin (g/dL) | 4.2 (2.8-5) |
| Baseline AST (IU/L) | 40 (14-197) |
| Baseline ALT (IU/L) | 40 (6-273) |
| Baseline γGTP (IU/L) | 33 (8-777) |
| Baseline Fib-4 index | 2.90 (0.54-82.8) |
| Baseline M2BPGi (C.O.I) | 2.04 (0.28-20) |
| Baseline AFP (ng/mL) | 4.6 (1.3-198.4) |
| Baseline T-C (mg/dL) | 171 (68-278) |
| Baseline HDL-C (mg/dL) | 51 (21-131) |
| Baseline LDL-C (mg/dL) | 93 (19-197) |
| Baseline Liver stiffness (kPa) | 6.8 (3.1-37.5) |
| Baseline CAP (dB/m) | 214 (100-343) |
| Baseline GA (%) | 22.1 (13.2-58.6) |
| Genotype (n=100) |  |
| MTP493 　GG/GT/TT | 65/29/5 |
| TM6SF2 　CC/CT/TT | 84/16/0 |
| PNPLA3 　CC/CG/GG | 30/41/16 |

Abbreviations: DCV, daclatasvir; ASV, Asunaprevir; SOF, sofosbuvir; LDV, ledipasvir; RBV, ribavirin; OBV, ombitasvir; PVT, paritaprevir; r, ritonavir; HCV, Hepatitis C virus; BMI, body mass index; ALT, alanine aminotransferase; AST, aspartate aminotransferase; γGTP, γ-glutamyl transpeptidase; M2BPGi, Mac-2 binding protein glycosylation isomer; AFP, alpha fetoprotein; T-C, total-cholesterol; HDL-C, high density lipoprotein-cholesterol; LDL-C, low density lipoprotein-cholesterol; CAP, Controlled Attenuation Parameter; GA, glycoalbumin. MTP493, microsomal triacylglycerol transfer protein 493; TM6SF2, transmembrane six superfamily member 2; PNPLA3, patatin-like phospholipase domain-containing protein 3.

^†^ Of 117, 100 patients were measured genotyping (In only MTP genotyping analysis, 99 patients were enrolled).

^‡^ Data are shown as median (range) values.
