# Supplementary material for: Liver steatosis and dyslipidemia after HCV eradication by direct acting antiviral agents are synergistic risks of atherosclerosis
Source: PLoS One. 2018 Dec 21;13(12):e0209615. doi: 10.1371/journal.pone.0209615 (PMC6303061; doi:10.1371/journal.pone.0209615)
Supplement: S2 Table — (DOCX) [file pone.0209615.s004.docx]

**Supplementary table 2**

**Comparison of baseline characteristics between patients with or without baseline CAP >220 dB/m**

|  | CAP <220 dB/m | CAP ≧220 dB/m | P value |
| --- | --- | --- | --- |
| Number | 63 | 54 |  |
| Age (years) | 66 (22-85) | 62.5 (35-83) | 0.407 |
| Sex (male/female) | 27/36 | 27/27 | 0.44 |
| HCV-RNA (log IU/mL) | 6.3 (3.2-7.1) | 6.25 (3.6-7.20) | 0.865 |
| BMI (kg/m^2^) | 21.4 (15.63-28.37) | 23.35 (16.63-30.86) | *0.004 |
| Baseline ALT (IU/L) | 37 (6-273) | 42.5 (11-262) | 0.333 |
| Baseline Fib-4 index | 3.01 (0.59-10.85) | 2.78 (0.54-82.8) | 0.498 |
| Baseline T-C (mg/dL) | 171 (68-253) | 171 (92-278) | 0.472 |
| Baseline HDL-C (mg/dL) | 51 (21-130) | 48.5 (22.6-110) | 0.531 |
| Baseline LDL-C (mg/dL) | 93 (19-197) | 95 (29-160) | 0.398 |
| Baseline Liver stiffness (kPa) | 6.9 (3.3-37.5) | 6.7 (3.1-27.7) | 0.573 |
| Baseline CAP (dB/m) | 181 (100-219) | 241.5 (220-343) | *<0.001 |
| Baseline GA (%) | 22.2 (13.2-52.6) | 21.1 (13.1-58.6) | 0.905 |
| Genotype: number (n=100) | 55 | 45 |  |
| MTP493　 GG/GT/TT | 36/15/4 | 29/14/1 | 0.739 |
| TM6SF2 CC/CT/TT | 46/9/0 | 38/7/0 | 0.913 |
| PNPLA3 CC/CG/GG | 21/24/10 | 14/21/10 | 0.739 |

Abbreviations: HCV, Hepatitis C virus; BMI, body mass index; ALT, alanine aminotransferase; T-C, total-cholesterol; HDL-C, high density lipoprotein-cholesterol; LDL-C, low density lipoprotein-cholesterol; CAP, controlled attenuation parameter; GA, glycoalbumin. MTP493, microsomal triacylglycerol transfer protein 493; TM6SF2, transmembrane six superfamily member 2; PNPLA3, patatin-like phospholipase domain-containing protein 3.

^†^ Data are shown as median (range) values.

*Statistically significant difference, P <0.05.
