# Supplementary material for: Liver steatosis and dyslipidemia after HCV eradication by direct acting antiviral agents are synergistic risks of atherosclerosis
Source: PLoS One. 2018 Dec 21;13(12):e0209615. doi: 10.1371/journal.pone.0209615 (PMC6303061; doi:10.1371/journal.pone.0209615)
Supplement: S3 Table — (DOCX) [file pone.0209615.s005.docx]

**Supplementary table 3**

**Comparison of baseline characteristics between patients with CAP > 220 dB/m that did or did not experience a decrease in CAP after HCV eradication**

|  | CAP value decrease | CAP value increase | P value |
| --- | --- | --- | --- |
| Number | 30 | 24 |  |
| Age (years) | 65.5 (41-83) | 61 (35-83) | 0.191 |
| Sex (male/female) | 14/16 | 13/11 | 0.584 |
| HCV-RNA (log IU/mL) | 6.2 (3.6-7.2) | 6.35 (4.7-7.1) | 0.185 |
| BMI (kg/m^2^) | 22.05 (16.63-29.96) | 24.34 (19.37-30.86) | *0.009 |
| Baseline ALT (IU/L) | 39.5 (13-211) | 60 (11-262) | 0.189 |
| Baseline Fib-4 index | 2.92 (0.54-82.8) | 2.23 (0.59-6.77) | 0.204 |
| Baseline T-C (mg/dL) | 164 (114-217) | 177 (92-278) | 0.052 |
| Baseline HDL-C (mg/dL) | 47.5 (22.6-102) | 50 (23-85) | 0.821 |
| Baseline LDL-C (mg/dL) | 82.5 (46-153) | 98.5 (29-160) | 0.112 |
| Baseline Liver stiffness (kPa) | 7.1 (3.1-26.3) | 5.25 (3.7-27.7) | 0.108 |
| Baseline CAP (dB/m) | 243 (223-343) | 237 (220-335) | 0.381 |
| Baseline GA (%) | 19.9 (14.9-46.3) | 24.95 (14.1-58.6) | 0.204 |
| Genotype: number (n=45) | 25 | 20 |  |
| MTP493　 GG/GT/TT | 19/5/0 | 10/9/1 | 0.1 |
| TM6SF2 CC/CT/TT | 22/3/0 | 16/4/0 | 0.371 |
| PNPLA3 　CC/CG/GG | 5/13/7 | 9/8/3 | 0.181 |

Abbreviations: HCV, Hepatitis C virus; BMI, body mass index; ALT, alanine aminotransferase; T-C, total-cholesterol; HDL-C, high density lipoprotein-cholesterol; LDL-C, low density lipoprotein-cholesterol; CAP, controlled attenuation parameter; GA, glycoalbumin. MTP493, microsomal triacylglycerol transfer protein 493; TM6SF2, transmembrane six superfamily member 2; PNPLA3, patatin-like phospholipase domain-containing protein 3.

^†^ Data are shown as median (range) values.
