# Supplementary material for: Liver steatosis and dyslipidemia after HCV eradication by direct acting antiviral agents are synergistic risks of atherosclerosis
Source: PLoS One. 2018 Dec 21;13(12):e0209615. doi: 10.1371/journal.pone.0209615 (PMC6303061; doi:10.1371/journal.pone.0209615)
Supplement: S4 Table — (DOCX) [file pone.0209615.s006.docx]

**Supplementary table 4**

**Comparison of baseline characteristics between patients with or without baseline LDL-C >108 mg/dL**

|  | LDL-C <108 mg/dL | LDL-C >108 mg/dL | P value |
| --- | --- | --- | --- |
| Number | 81 | 36 |  |
| Age (years) | 65 (22-83) | 62 (35-85) | 0.645 |
| Sex (male/female) | 38/43 | 16/20 | 0.805 |
| HCV-RNA (log IU/mL) | 6.3 (3.2-7.2) | 6.3 (3.6-6.9) | 0.586 |
| BMI (kg/m^2^) | 21.67 (15.63-30.86) | 23.05 (18.47-30.73) | 0.119 |
| Baseline ALT (IU/L) | 40 (11-262) | 40.5 (6-273) | 0.48 |
| Baseline Fib-4 index | 2.93 (0.54-82.8) | 2.32 (0.59-13.51) | 0.173 |
| Baseline T-C (mg/dL) | 158 (68-234) | 203 (165-278) | *<0.001 |
| Baseline HDL-C (mg/dL) | 52 (21.8-111.8) | 54.2 (23-110) | 0.301 |
| Baseline LDL-C (mg/dL) | 75 (19-107) | 122.5 (108-197) | *<0.001 |
| Baseline Liver stiffness (kPa) | 6.9 (3.3-35.3) | 6.05 (3.1-37.5) | 0.2 |
| Baseline CAP (dB/m) | 212 (100-343) | 214.5 (102-335) | 0.962 |
| Baseline GA (%) | 21.35 (13.2-58.6) | 23.9 (14.9-52.6) | 0.057 |
| Genotype: number (n=100) | 71 | 29 |  |
| MTP493　 GG/GT/TT | 42/24/4 | 23/5/1 | 0.182 |
| TM6SF2 CC/CT/TT | 59/12/0 | 25/4/0 | 0.478 |
| PNPLA3 CC/CG/GG | 23/33/15 | 12/12/5 | 0.688 |

Abbreviations: HCV, Hepatitis C virus; BMI, body mass index; ALT, alanine aminotransferase; T-C, total-cholesterol; HDL-C, high density lipoprotein-cholesterol; LDL-C, low density lipoprotein-cholesterol; CAP, controlled attenuation parameter; GA, glycoalbumin. MTP493, microsomal triacylglycerol transfer protein 493; TM6SF2, transmembrane six superfamily member 2; PNPLA3, patatin-like phospholipase domain-containing protein 3.

^†^ Data are shown as median (range) values.

*Statistically significant difference, P <0.05.
