# Supplementary material for: Liver steatosis and dyslipidemia after HCV eradication by direct acting antiviral agents are synergistic risks of atherosclerosis
Source: PLoS One. 2018 Dec 21;13(12):e0209615. doi: 10.1371/journal.pone.0209615 (PMC6303061; doi:10.1371/journal.pone.0209615)
Supplement: S5 Table — (DOCX) [file pone.0209615.s007.docx]

**Supplementary table 5**

**Comparison of baseline characteristics between patients with LDL-C >108 dB/m that did or did not experience a decrease in LDL-C after HCV eradication**

|  | LDL-C level decrease | LDL-C level increase | P value |
| --- | --- | --- | --- |
| Number | 16 | 20 |  |
| Age (years) | 63.5 (41-85) | 62 (35-83) | 0.498 |
| Sex (male/female) | 5/11 | 11/9 | 0.154 |
| HCV-RNA (log IU/mL) | 6.3 (3.6-6.9) | 6.1 (4.5-6.8) | 0.404 |
| BMI (kg/m^2^) | 22.08 (18.47-28.3) | 23.74 (18.97-30.73) | *0.034 |
| Baseline ALT (IU/L) | 28 (13-273) | 52.5 (6-179) | 0.223 |
| Baseline Fib-4 index | 2.49 (0.73-9.12) | 1.86 (0.59-13.51) | 0.2 |
| Baseline T-C (mg/dL) | 209.5 (165-253) | 193 (170-278) | 0.158 |
| Baseline HDL-C (mg/dL) | 53 (24.8-100.4) | 55.2 (23-110) | 1 |
| Baseline LDL-C (mg/dL) | 127.5 (114-197) | 117.5 (108-160) | *0.039 |
| Baseline Liver stiffness (kPa) | 5.7 (3.1-37.5) | 6.8 (3.3-27.7) | 0.352 |
| Baseline CAP (dB/m) | 206.5 (151-295) | 219 (102-335) | 0.32 |
| Baseline GA (%) | 20.6 (14.9-52.6) | 25.6 (17.8-46.6) | 0.276 |
| Genotype: number (n=100) | 13 | 16 |  |
| MTP493　 GG/GT/TT | 12/1/0 | 11/3/1 | 0.278 |
| TM6SF2 CC/CT/TT | 12/1/0 | 13/3/0 | 0.383 |
| PNPLA3 CC/CG/GG | 5/6/2 | 7/6/3 | 0.893 |

Abbreviations: HCV, Hepatitis C virus; BMI, body mass index; ALT, alanine aminotransferase; T-C, total-cholesterol; HDL-C, high density lipoprotein-cholesterol; LDL-C, low density lipoprotein-cholesterol; CAP, controlled attenuation parameter; GA, glycoalbumin. MTP493, microsomal triacylglycerol transfer protein 493; TM6SF2, transmembrane six superfamily member 2; PNPLA3, patatin-like phospholipase domain-containing protein 3.

^†^ Data are shown as median (range) values.

*Statistically significant difference, P <0.05.
