# Supplementary material for: Liver steatosis and dyslipidemia after HCV eradication by direct acting antiviral agents are synergistic risks of atherosclerosis
Source: PLoS One. 2018 Dec 21;13(12):e0209615. doi: 10.1371/journal.pone.0209615 (PMC6303061; doi:10.1371/journal.pone.0209615)
Supplement: S6 Table — (DOCX) [file pone.0209615.s008.docx]

**Supplementary table 6**

**Comparison of baseline characteristics between patients with CAP <220 dB/m that did or did not exceed CAP >248 dB/m at SVR24 point**

|  | CAP <248 dB/m | CAP value ≧248 dB/m | P value |
| --- | --- | --- | --- |
| Number | 51 | 12 |  |
| Age (years) | 66 (22-85) | 62 (46-82) | 0.482 |
| Sex (male/female) | 21/30 | 6/6 | 0.578 |
| HCV-RNA (log IU/mL) | 6.3 (4.2-71) | 6.35 (3.2-6.8) | 0.93 |
| BMI (kg/m^2^) | 20.39 (15.63-28.3) | 25.09 (20.32-28.37) | *<0.001 |
| Baseline ALT (IU/L) | 36 (6-273) | 51 (15-71) | 0.363 |
| Baseline Fib-4 index | 3.06 (0.59-10.85) | 2.76 (1.25-9.12) | 0.739 |
| Baseline T-C (mg/dL) | 171 (68-253) | 156 (94-247) | 0.972 |
| Baseline HDL-C (mg/dL) | 53 (21-131) | 32 (22-71) | *0.012 |
| Baseline LDL-C (mg/dL) | 93 (19-143) | 100 (40-197) | 0.259 |
| Baseline Liver stiffness (kPa) | 6.8 (3.3-25.4) | 7.7 (4.5-37.5) | 0.462 |
| Baseline CAP (dB/m) | 179 (100-219) | 197 (162-216) | 0.07 |
| Baseline GA (%) | 22.7 (13.2-52.6) | 20.3 (14.8-50.9) | 0.274 |
| Genotype: number (n=100) | 45 | 10 |  |
| MTP493　 GG/GT/TT | 29/14/2 | 7/1/2 | 0.129 |
| TM6SF2 CC/CT/TT | 36/9/0 | 10/0/0 | 0.139 |
| PNPLA3 CC/CG/GG | 15/20/10 | 6/4/0 | 0.151 |

Abbreviations: HCV, Hepatitis C virus; BMI, body mass index; ALT, alanine aminotransferase; T-C, total-cholesterol; HDL-C, high density lipoprotein-cholesterol; LDL-C, low density lipoprotein-cholesterol; CAP, controlled attenuation parameter; GA, glycoalbumin. MTP493, microsomal triacylglycerol transfer protein 493; TM6SF2, transmembrane six superfamily member 2; PNPLA3, patatin-like phospholipase domain-containing protein 3.

^†^ Data are shown as median (range) values.

*Statistically significant difference, P <0.05.
