# Supplementary material for: Liver steatosis and dyslipidemia after HCV eradication by direct acting antiviral agents are synergistic risks of atherosclerosis
Source: PLoS One. 2018 Dec 21;13(12):e0209615. doi: 10.1371/journal.pone.0209615 (PMC6303061; doi:10.1371/journal.pone.0209615)
Supplement: S7 Table — (DOCX) [file pone.0209615.s009.docx]

**Supplementary table 7**

**Association among changes in clinical parameters after HCV eradication according to the PNPLA3 genetic polymorphisms**

| PNPLA3 | CC | CG | GG | P value |
| --- | --- | --- | --- | --- |
| Number | 35 | 45 | 20 |  |
| Baseline CAP value (dB/m) | 210 (102-335) | 215 (100-302) | 215 (103-343) | 0.976 |
| CAP value at SVR24 (dB/m) | 225 (147-362) | 216 (144-306) | 212 (122-308) | 0.305 |
| Baseline T-C (mg/dL) | 171 (68-278) | 171 (94-247) | 169 (117.4-253) | 0.56 |
| T-C at SVR24 (mg/dL) | 205 (115-237) | 194 (127-253) | 190.5 (130-244) | 0.374 |
| Baseline LDL-C (mg/dL) | 86 (19-147) | 93 (43-197) | 85 (46-153) | 0.841 |
| LDL-C at SVR24 (mg/dL) | 107 (10-182) | 112 (56-162) | 100.5 (64-160) | 0.709 |
| Baseline HDL-C (mg/dL) | 47 (21-110) | 54 (24.8-102) | 49.5 (22.6-131) | 0.159 |
| HDL-C at SVR24 (mg/dL) | 52 (20.6-105) | 56 (31-96) | 59.2 (19.8-101.2) | 0.246 |
| Baseline Liver stiffness (kPa) | 6.1 (3.4-37.5) | 6.9 (3.1-27.7) | 6.15 (3.3-27.7) | 0.914 |
| Liver stiffness at SVR24 (kPa) | 5.4 (2.3-50.5) | 5.5 (2.6-21.3) | 5.1 (3.3-48) | 0.601 |
| Baseline GA (%) | 22.6 (14.3-52.6) | 20.45 (13.2-46.3) | 22.45 (13.5-49.3) | 0.241 |
| GA at SVR24 (%) | 16.3 (10.6-28.9) | 16.1 (11.9-34.4) | 15.5 (12.4-35.6) | 0.997 |

Abbreviations: PNPLA3, patatin-like phospholipase domain-containing protein 3; CAP, controlled attenuation parameter; T-C, total- cholesterol; LDL-C, low density lipoprotein-cholesterol; HDL-C, high density lipoprotein-cholesterol; LS, liver stiffness; GA, glycoalbumin.

^†^ Of 117, 100 patients were measured genotyping.

^‡^ Data are shown as median (range) values.

*Statistically significant difference, P <0.05.
