# Supplementary material for: Liver steatosis and dyslipidemia after HCV eradication by direct acting antiviral agents are synergistic risks of atherosclerosis
Source: PLoS One. 2018 Dec 21;13(12):e0209615. doi: 10.1371/journal.pone.0209615 (PMC6303061; doi:10.1371/journal.pone.0209615)
Supplement: S8 Table — (DOCX) [file pone.0209615.s010.docx]

**Supplementary table 8**

**Association among changes in clinical parameters after HCV eradication according to the TM6SF2 genetic polymorphisms**

| TM6SF2 | CC | CT+TT | P value |
| --- | --- | --- | --- |
| Number | 84 | 16/0 |  |
| Baseline CAP value (dB/m) | 213 (102-343) | 208 (100-335) | 0.632 |
| CAP value at SVR24 (dB/m) | 218 (122-362) | 219 (168-356) | 0.714 |
| Baseline T-C (mg/dL) | 170.5 (68-278) | 173.5 (112-253) | 0.985 |
| T-C at SVR24 (mg/dL) | 193 (115-253) | 205 (130-250) | 0.507 |
| Baseline LDL-C (mg/dL) | 91.5 (19-197) | 93 (46-123) | 0.408 |
| LDL-C at SVR24 (mg/dL) | 109 (10-182) | 106 (64-172) | 0.595 |
| Baseline HDL-C (mg/dL) | 51 (21-131) | 54 (37-100.4) | 0.267 |
| HDL-C at SVR24 (mg/dL) | 53.9 (19.8-1059 | 58 (34.8-95) | 0.535 |
| Baseline Liver stiffness (kPa) | 6.8 (3.1-37.5) | 8.6 (3.3-27.7) | 0.405 |
| Liver stiffness at SVR24 (kPa) | 5.35 (2.3-50.5) | 6.4 (3.3-48) | 0.098 |
| Baseline GA (%) | 21.2 (13.2-52.6) | 22.1 (15.9-46.6) | 0.252 |
| GA at SVR24 (%) | 17.3 (11.4-38.9) | 18.55 (13.2-27) | 0.297 |

Abbreviations: TM6SF2, transmembrane six superfamily member 2; CAP, controlled attenuation parameter; T-C, total-cholesterol; LDL-C, low density lipoprotein-cholesterol; HDL-C, high density lipoprotein-cholesterol; LS, liver stiffness; GA, glycoalbumin.

^†^ Of 117, 100 patients were measured genotyping.

^‡^ Data are shown as median (range) values.

*Statistically significant difference, P <0.05.
