# Supplementary material for: Liver steatosis and dyslipidemia after HCV eradication by direct acting antiviral agents are synergistic risks of atherosclerosis
Source: PLoS One. 2018 Dec 21;13(12):e0209615. doi: 10.1371/journal.pone.0209615 (PMC6303061; doi:10.1371/journal.pone.0209615)
Supplement: S9 Table — (DOCX) [file pone.0209615.s011.docx]

**Supplementary table 9**

**Association among changes in clinical parameters after HCV eradication according to the MTP-493 genetic polymorphisms**

| MTP493 | GG | GT+TT | P value |
| --- | --- | --- | --- |
| Number | 65 | 29/5 |  |
| Baseline CAP value (dB/m) | 211 (100-338) | 211 (115-343) | 0.938 |
| CAP value at SVR24 (dB/m) | 218 (147-339) | 220 (122-362) | 0.477 |
| Baseline T-C (mg/dL) | 172.2 (90-278) | 161.2 (68-217) | *0.023 |
| T-C at SVR24 (mg/dL) | 193.2 (130-250) | 193 (115-253) | 0.5 |
| Baseline LDL-C (mg/dL) | 95 (40-197) | 78.5 (19-134.8) | 0.074 |
| LDL-C at SVR24 (mg/dL) | 107 (64-182) | 104 (10-160) | 0.256 |
| Baseline HDL-C (mg/dL) | 53 (23.6-131) | 48.5 (21-84) | 0.165 |
| HDL-C at SVR24 (mg/dL) | 55 (19.8-101.2) | 55 (26-105) | 0.927 |
| Baseline Liver stiffness (kPa) | 6.7 (3.1-37.5) | 6.85 (3.3-27.7) | 0.962 |
| Liver stiffness at SVR24 (kPa) | 5.4 (2.3-50.5) | 5.5 (2.6-48) | 0.802 |
| Baseline GA (%) | 21.15 (13.2-52.6) | 22.45 (14.1-49.3) | 0.791 |
| GA at SVR24 (%) | 15.8 (10.6-28.9) | 17.6 (11.9-35.6) | 0.143 |

Abbreviations: MTP-493, microsomal triacylglycerol transfer protein 493; CAP, controlled attenuation parameter; T-C, total-cholesterol; LDL-C, low density lipoprotein-cholesterol; HDL-C, high density lipoprotein-cholesterol; LS, liver stiffness; GA, glycoalbumin.

^†^ Of 117, 99 patients were measured genotyping.

^‡^ Data are shown as median (range) values.

*Statistically significant difference, P <0.05.
